# Brain miliary enhancement

**DOI:** 10.1007/s00234-019-02335-5

**Published:** 2020-01-10

**Authors:** Joseph C.J. Bot, Linda Mazzai, Rogier E. Hagenbeek, Silvia Ingala, Bob van Oosten, Esther Sanchez-Aliaga, Frederik Barkhof

**Affiliations:** 1 Department of Radiology and Nuclear Medicine, Amsterdam UMC, Location VUmc, P.O. Box 7057, 1007 MB Amsterdam, The Netherlands; 2 Institute of Radiology, Department of Medicine (DiMED), University of Padua, Padua, Italy; 3 Haaglanden Medisch Centrum, The Hague, The Netherlands; 4 Department of Neurology, Amsterdam UMC, Location VUmc, Amsterdam, The Netherlands; 5 Institute of Neurology and Healthcare Engineering, UCL, London, UK

**Keywords:** Magnetic resonance imaging, Brain, Contrast, Miliary, Virchow–Robin spaces

## Abstract

**Purpose:**

Miliary enhancement refers to the presence of multiple small, monomorphic, enhancing foci on T1-weighted post-contrast MRI images. In the absence of a clear clinical presentation, a broad differential diagnosis may result in invasive procedures and possibly brain biopsy for diagnostic purposes.

**Methods:**

An extensive review of the literature is provided for diseases that may present with miliary enhancement on T1-weighted brain MR images. Additional disease-specific findings, both clinical and radiological, are summarized and categorized by the presence or absence of perivascular space involvement.

**Results:**

Miliary pattern of enhancement may be due to a variety of underlying causes, including inflammatory, infectious, nutritional or neoplastic processes. The recognition of disease spread along the perivascular spaces in addition to the detection or exclusion of disease-specific features on MRI images, such as leptomeningeal enhancement, presence of haemorrhagic lesions, spinal cord involvement and specific localisation or systemic involvement, allows to narrow the potential differential diagnoses.

**Conclusion:**

A systematic approach to disease-specific findings from both clinical and radiological perspectives might facilitate diagnostic work-up, and recognition of disease spread along the perivascular spaces may help narrowing down differential diagnoses and may help to minimize the use of invasive diagnostic procedures.

## Introduction

Contrast-enhanced MRI of the CNS is routinely used in clinical practice. Parenchymal enhancement after intravenous contrast administration is not disease-specific and may therefore be associated with many pathological processes, as it reflects either the breakdown of the blood–brain barrier or the presence of one or more lesions within or surrounding the vessels [1]. Depending on the pathological substrate and anatomical structure, the patterns of enhancement, as seen on post-gadolinium T1-weighted images, may vary or mimic each other. Miliary refers to the size of a millet seed, and it is generally used to describe a pattern of multiple punctate uniform enhancements with monomorphic dot-like lesions with a diameter smaller than 3 mm [2]. The designation miliary is descriptive, non-specific with respect to aetiology, and it may be due to a variety of underlying conditions, ranging from inflammatory, infectious, nutritional to neoplastic processes. When confronted with a non-specific clinical presentation and such a pattern of lesion appearance on MRI, a broad differential diagnosis for the neuroimaging findings remains. A systematic approach in diagnostic work-up could prevent unnecessary brain biopsy. To our knowledge, only one previously published report has addressed this miliary enhancement pattern of small vessel lumen or its perivascular areas in case of blood–brain barrier disruption [2], describing punctate and linear enhancing brain lesions within three different pathological conditions. In this paper, we describe a more extensive differential diagnosis of diseases and elaborate also on diseases that may present with miliary enhancement outside the perivascular spaces (PVS).

Firstly, the medical history and physical examination should pay attention to less prominent symptoms of systemic disease, and laboratory findings, especially CSF analysis, should be scrutinized.

**Fig. 1:**
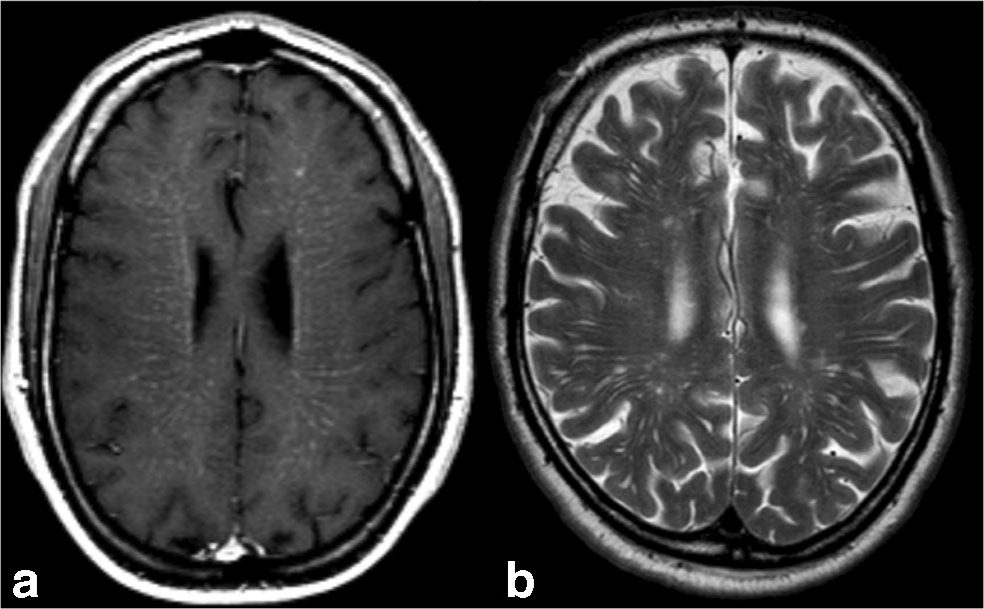
**a** Axial post-contrast T1-weighted MRI at the level of the corona radiata in a 39-years-old male affected by vitamin B_12_ deficiency. Note the miliary-enhancing nodules in addition to linear enhancement along the pathway of perforating vessels, suggestive for PVS involvements. In this patient, brain abnormalities totally resolved after vitamin B_12_ suppletion therapy. **b** Note on a T2-weighted MRI in a different and healthy subject the T2 hyperintense prominent PVS identical to the enhancement pattern

Secondly, the miliary enhancement pattern on MRI should be analysed to determine whether distribution along the PVS, also known as Virchow–Robin spaces, is present (Fig. 1). The PVS are pial-lined interstitial fluid-filled spaces in the brain that surround perforating vessels positioned along the lenticulostriate arteries entering the basal ganglia, along the medullary arteries over the high convexities and at the level of the mesencephalon-diencephalic junction [3]. This intracerebral compartment drains interstitial fluid and communicates with CSF spaces through active transport mechanisms. PVS are non-enhancing after the intravenous administration of gadolinium-based contrast agent. In some disorders, miliary-enhancing brain lesions may be seen along the PVS with or without additional linear enhancement, while others do not (Table 1, Fig. 1).

**Table 1:** Differential diagnosis of miliary enhancing brain lesions according to PVS involvement

PVS involvement	Non-PVS involvement
Metastatic disease	Metastatic disease
Infectious diseases: Lyme’s disease, Candida albicans, Cryptococcus, PML-IRIS	Infectious diseases: Tuberculosis, Histoplasmosis
CNS lymphoma	Susac’s syndrome
Neurosarcoidosis	
CNS vasculitis	
Erdheim Chester’s Disease	
Vitamin B12 deficiency	
CLIPPERS	
Behçet’s disease	
GFAP meningoencephalomyelitis	
NMOSD	

**Fig. 2:**
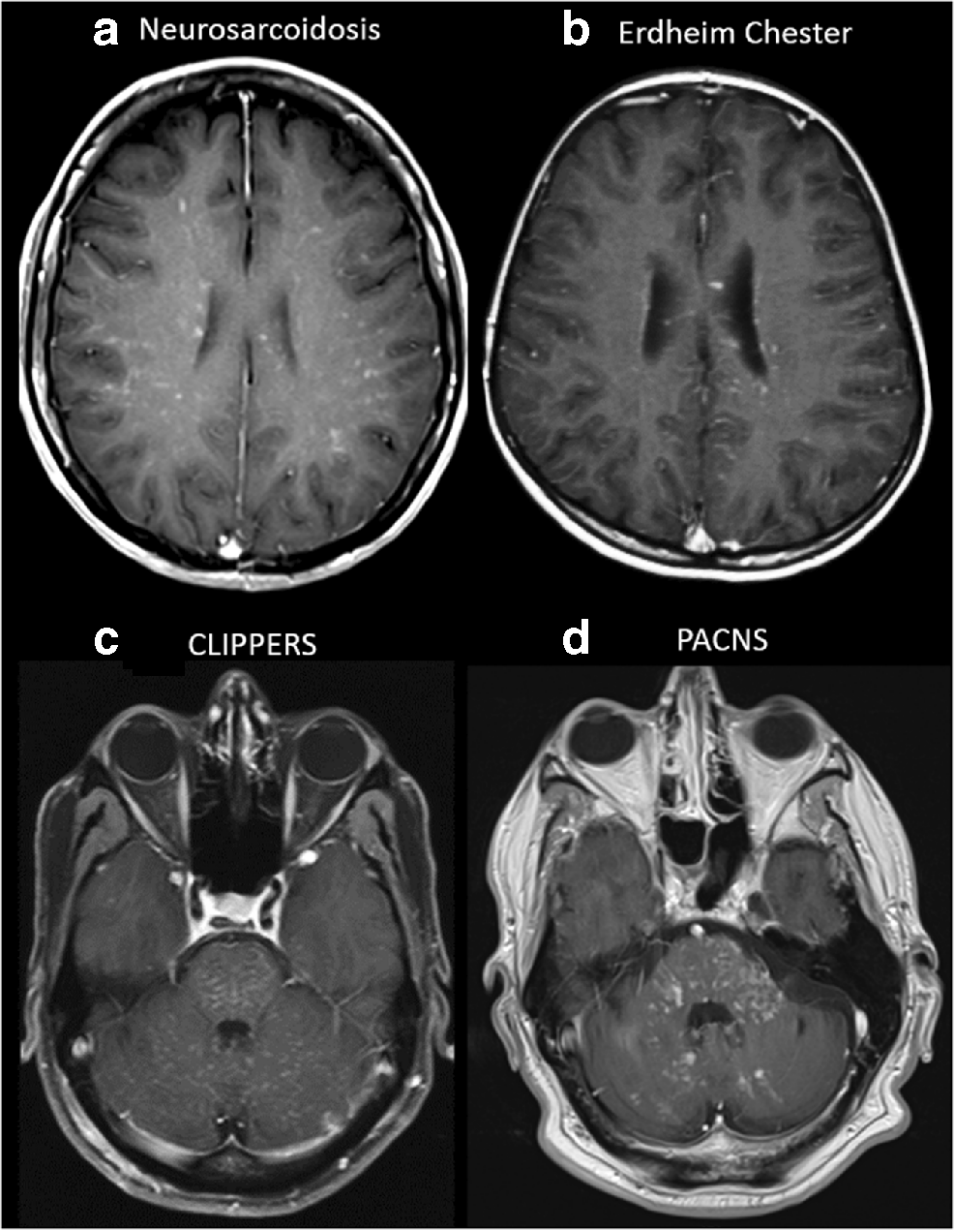
Examples of diseases with brain miliary enhancement with PVS pattern. **a**, **b** Axial post-contrast T1-weighted MRI at the level of the corona radiata. **a** A 53-year-old female with skin biopsy–proven neurosarcoidosis. **b** A 24-year-old female affected by Erdheim–Chester disease. Both patients show miliary-enhancing nodules scattered in the brain parenchyma with a PVS pattern along the course of perforating vessels. **c**, **d** Axial post-contrast T1-weighted MRI at the level of the posterior fossa. Please note the different orientations of the PVS in the brainstem and cerebellum. **c** A 55-year-old male with CLIPPERS with miliary enhancement, ‘peppering’ the pons, with spread to the cerebellum and subtle linear enhancement along the PVS. Brain abnormalities totally resolved in this case after corticosteroid therapy. **d** A 57-year-old male with PACNS shows miliary enhancement in the pons, cerebellar peduncles and cerebellar hemispheres. Angiography (not shown) was unremarkable in this brain biopsy–proven case of primary CNS angiitis

**Fig. 3:**
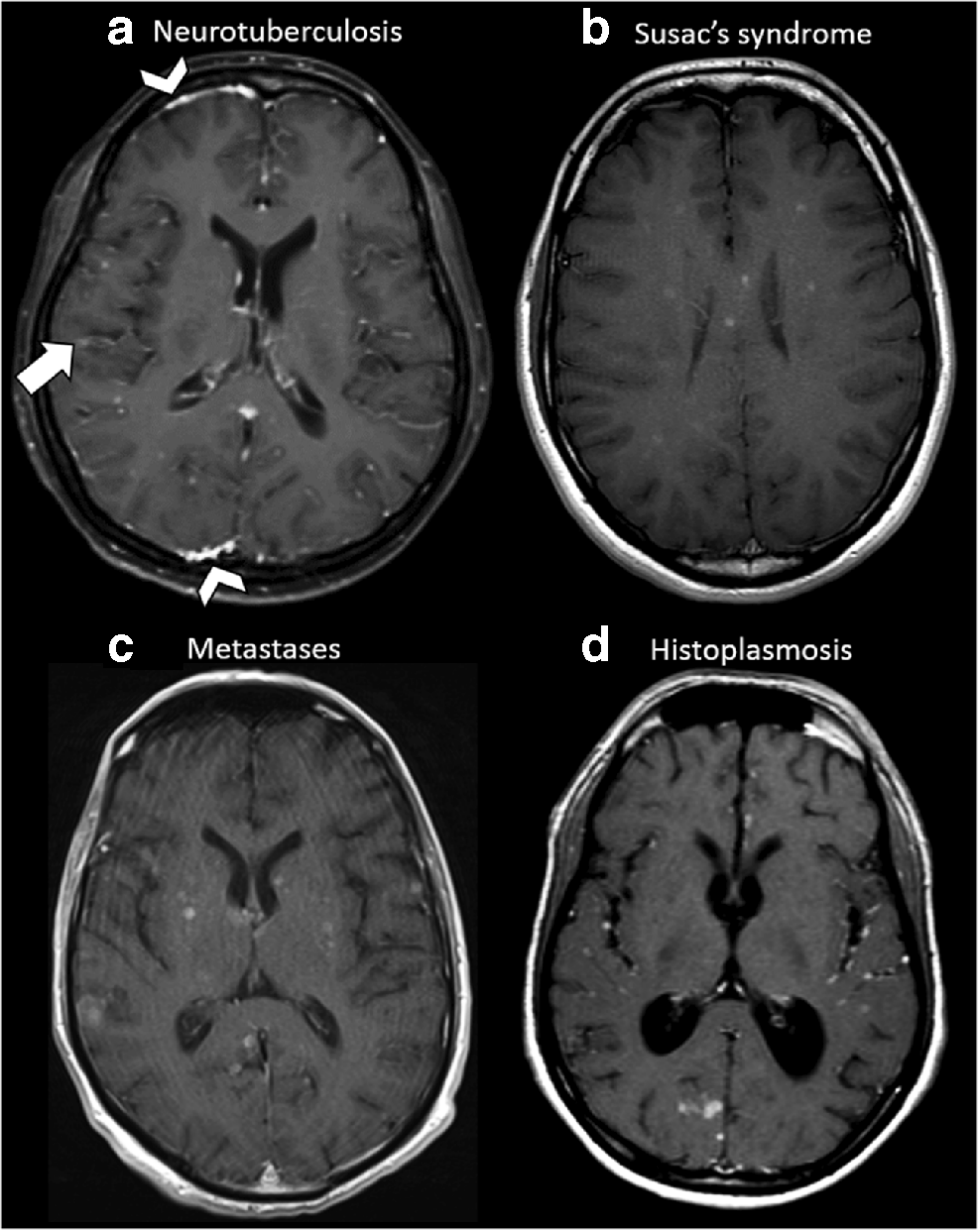
Examples of diseases with brain miliary enhancement without PVS pattern. Axial post-contrast T1-weighted MRI of the supratentorial region. **a** A 35-year-old female with neurotuberculosis (proven by bronchoalveolar lavage culture). Multiple miliary-enhancing nodules are visible at the white–grey matter junction. Note the faint leptomeningeal enhancement visible at the right parietal operculum (arrow) and the pachymeningeal involvement visible in this slice in the frontal and occipital right lobes (arrowheads). **b** A 20-year-old female affected by Susac’s syndrome. Note the typical corpus callosum involvement and small enhancing nodules without PVS distribution are scattered through the supratentorial brain. **c** A 72-year-old male with lung adenocarcinoma. Multiple miliary-enhancing nodules are detectable at the level of the basal ganglia, right temporal and occipital lobes and left parietal operculum in random distribution without clear PVS distribution. Please also note the slight variability size of nodules. **d** A 27-year-old male from Central America with lung biopsy–proven histoplasmosis. Multiple miliary-enhancing nodules are visible at the grey–white matter junction and in the cortex, besides few bigger enhancing nodules in the occipital right lobe. Leptomeningeal enhancement is recognizable in the left insula and left parietal operculum. Note the enlargement of the ventricle system, caused by secondary hydrocephalus due to basilar leptomeningeal involvement (not shown in this slice)

**Fig. 4:**
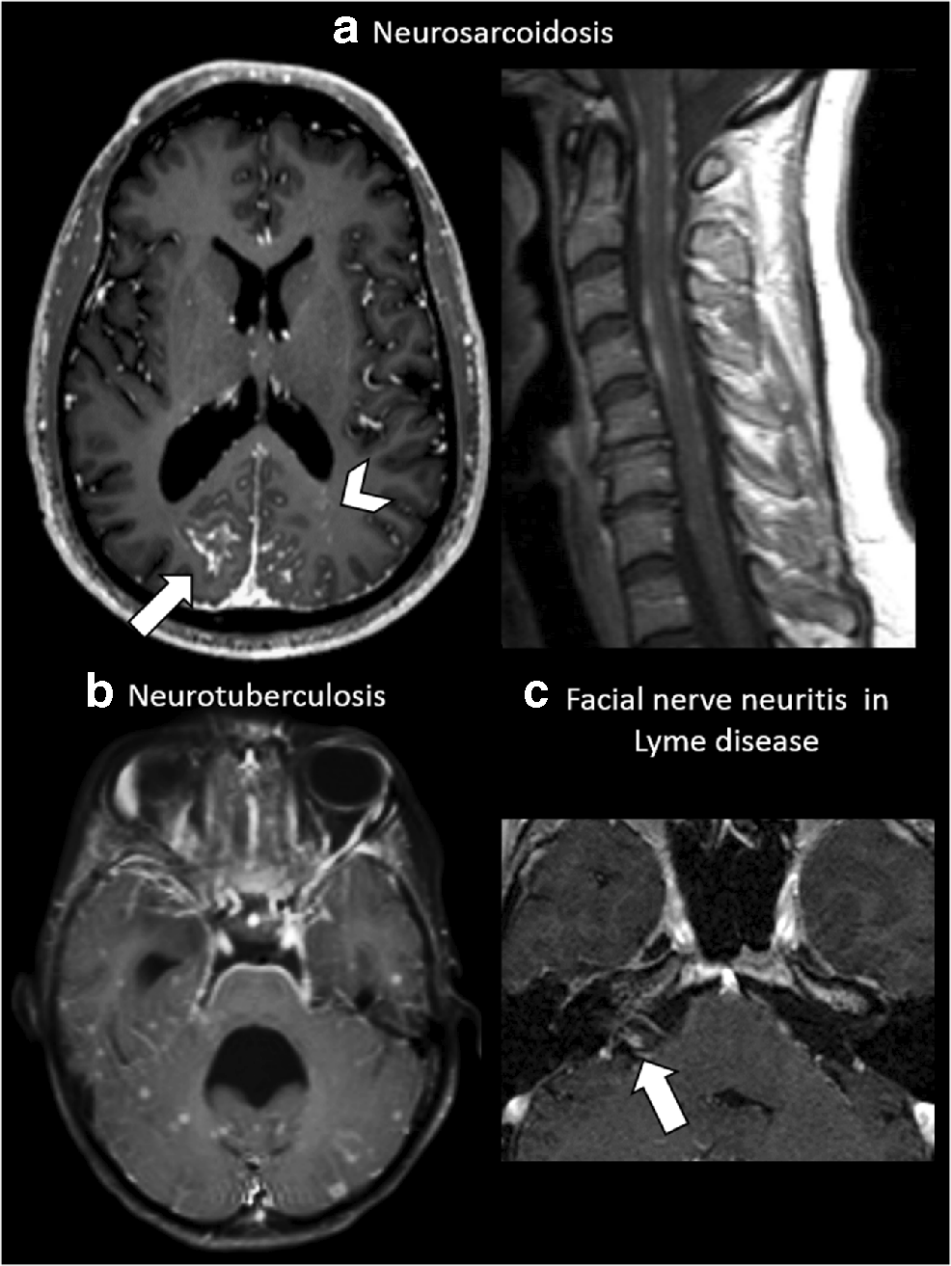
Leptomeningeal enhancement. **a** A 35-year-old male affected by skin biopsy–proven neurosarcoidosis. Axial post-contrast T1-weighted MRI at the level of the basal ganglia shows extensive parietal leptomeningeal enhancement (arrow), more evident on the right side, with multiple miliary-enhancing nodules with a distribution pattern suggestive of PVS involvement in the left occipital lobe (arrowhead). The same patient also shows extensive irregular leptomeningeal enhancement at the level of the cervical spinal cord. **b** A 26-year-old female with neurotuberculosis. Axial post-contrast T1-weighted MRI at the level of the 4th ventricle demonstrates an extensive leptomeningeal involvement at the ventral pons, fifth cranial nerves (arrows) and the petroclinoid ligaments. Note the non-PVS pattern of the miliary nodules scattered at random throughout the temporal lobes and cerebellum. Note the 4th ventricle dilatation, caused by secondary hydrocephalus due to the extensive basilar leptomeningeal involvement. **c** A 37-year-old female with cranial nerve VII neuritis due to Lyme disease. Axial magnification of the cerebellopontine angle post-contrast T1-weighted MRI shows intense contrast enhancement of the cisternal segment of the right facial nerve (arrow)

**Fig. 5:**
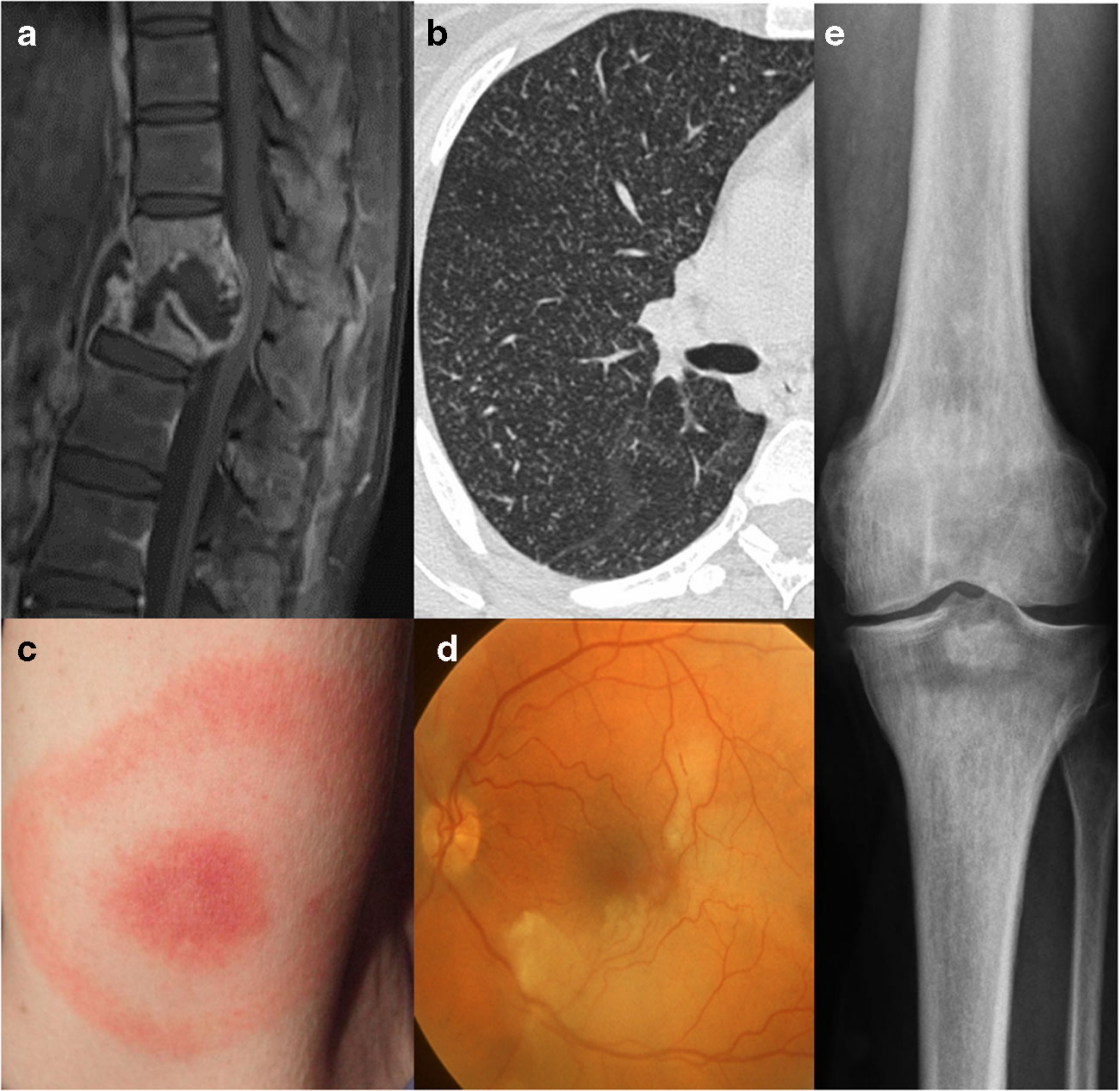
Systemic involvement. **a** A 26-year-old female with neurotuberculosis. Sagittal post-contrast T1-weighted MRI with fat saturation shows collapse of thoracic vertebrae, destruction of the interposed vertebral disc, prevertebral and intervertebral fluid collection with peripheral enhancing margins and diffusely enhancing remainder of the thoracic vertebrae, suggesting spondylodiscitis with abscess formation, known as Pott’s disease. Due to displacement and mass effect narrowing of the spinal canal with compression of the spinal cord. **b** Axial high-resolution CT scan (right lung) in the same patient showing ‘miliary tuberculosis’ with innumerable small pulmonary nodules with centrilobular distribution. **c** A 37-year-old female with a typical erythema migrans, as a disease-specific finding in Lyme disease. **d** A 20-year-old female affected by Susac’s syndrome with branch retinal artery occlusion. Fundus photography of the left eye shows ischaemic retinal oedema and narrowed branches of the superior and inferior temporal arteries. **e** A 24-year-old female affected by Erdheim–Chester disease with a sclerotic metaphyseal lesion in the tibia as a disease-specific finding

**Fig. 6:**
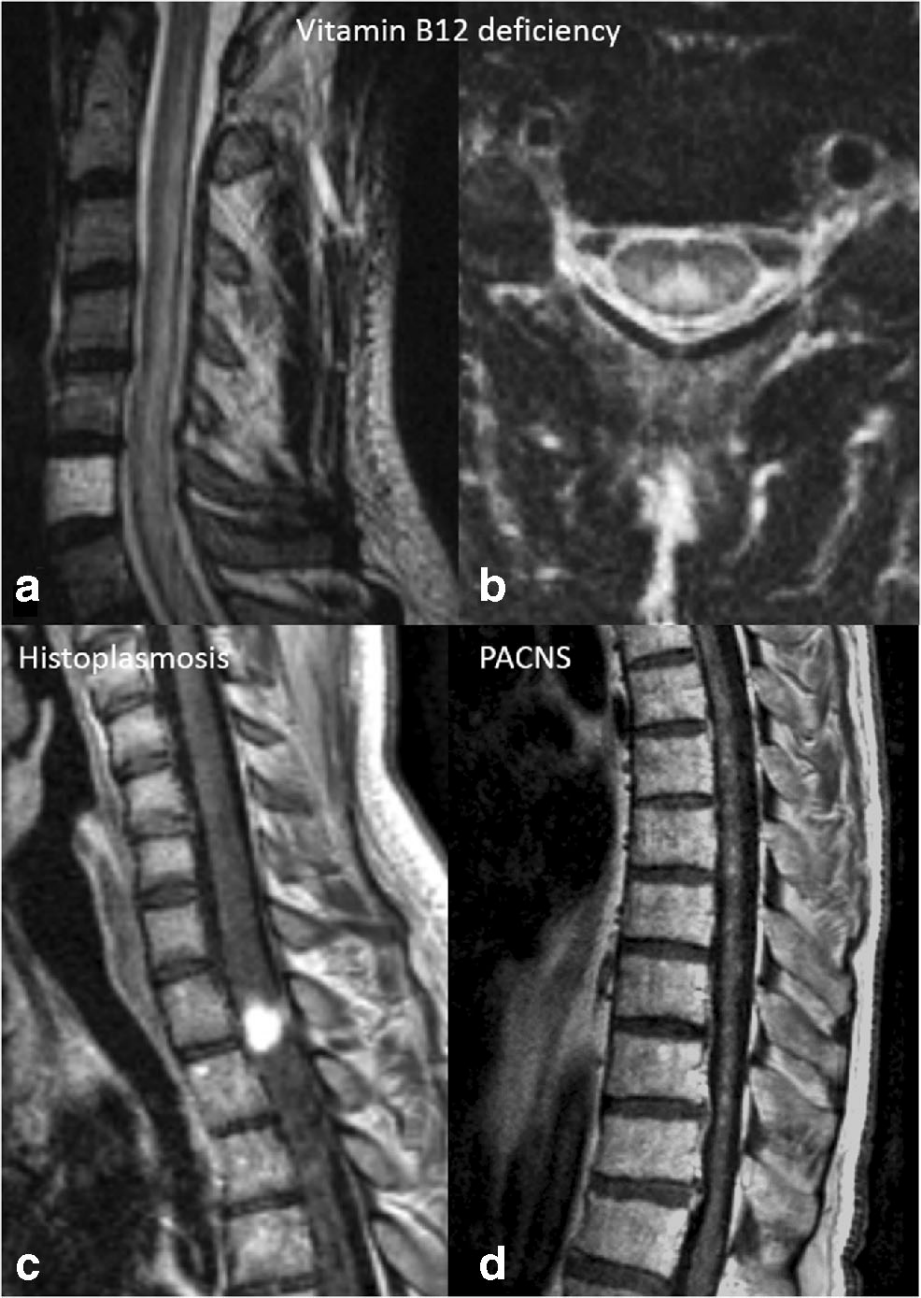
Different patterns of spinal cord involvement. A 39-year-old male with vitamin B_12_ deficiency (see also Fig. 1 for brain images). **a** Sagittal T2-weighted MRI of the cervical spine showing an extensive multisegmental involvement of the dorsal column of the spinal cord without obvious swelling. **b** Axial T2-weighted MRI of the cervical spine in the same patient clearly shows the dorsal column involvement of the spinal cord, as a typical finding in vitamin B_12_ deficiency. **c** Sagittal post-contrast T1-weighted MRI of the cervical and thoracic spine in a 27-year-old male from Central America with lung biopsy–proven histoplasmosis shows a nodular enhancement in the spinal cord at the C7–T1 level. **d** Sagittal post-contrast T1-weighted MRI of the thoracic spine in a 57-year-old male with brain biopsy–proven PACNS shows a miliary pattern of intramedullary spinal cord enhancement

**Fig. 7:**
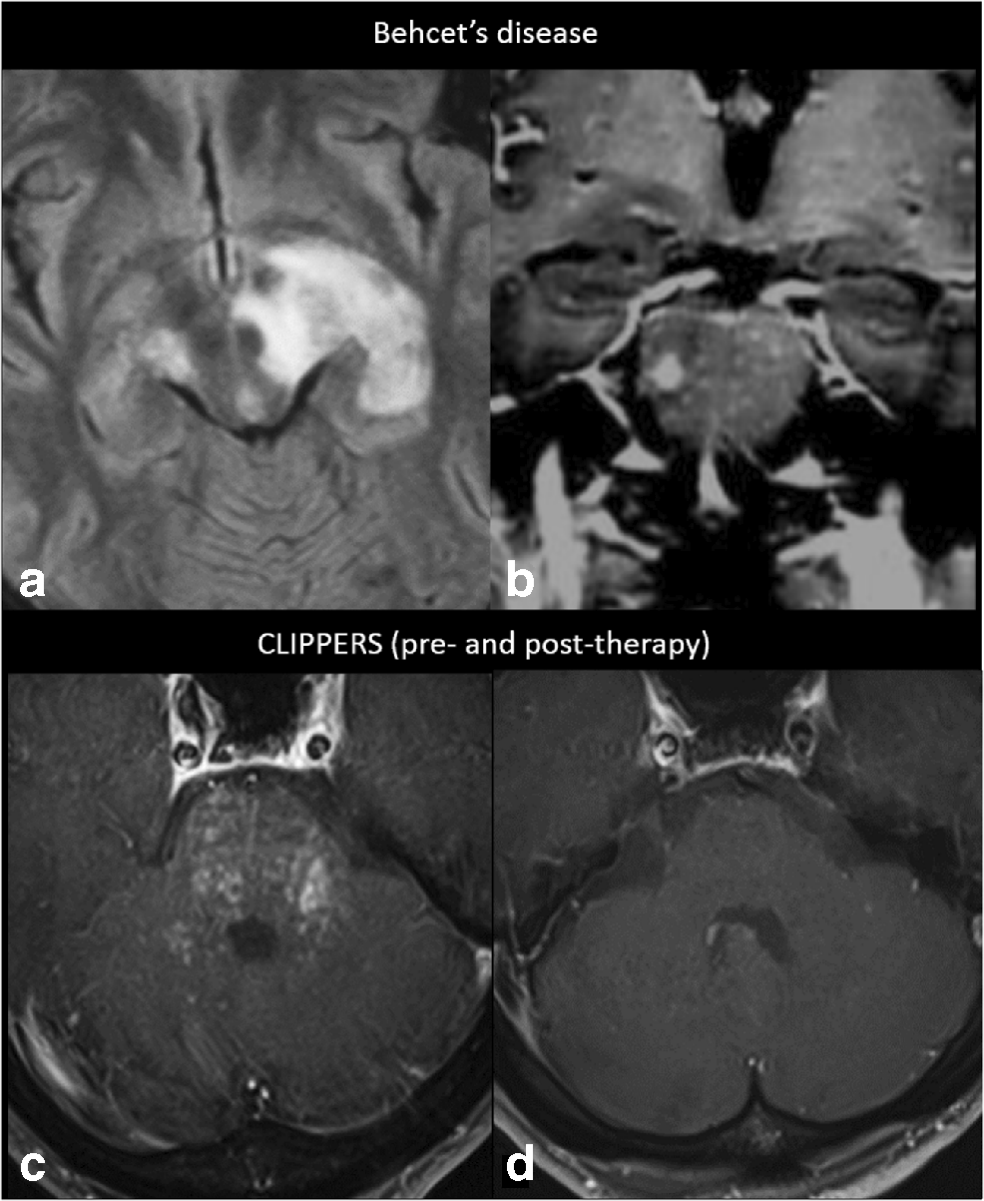
Preferentially infratentorial localisation. A 35-year-old male diagnosed with Behçet’s disease (small intestine biopsy proven) shows in **a** axial FLAIR MRI at the level of the crus cerebri a heterogeneous left mesodiencephalic lesion with oedema, typically sparing the red nucleus, and in **b** coronal post-contrast T1-weighted MRI at the level of the pons miliary enhancement and one larger nodule. **c** Axial post-contrast T1-weighted MRI of a 55-year-old male diagnosed with CLIPPERS, showing miliary-enhancing nodules and slight linear enhancement along the PVS in the pons, dentate nuclei and spreading through the cerebellum and a **d** complete resolution of the infratentorial lesions after corticosteroid therapy

It is hard to distinguish the aetiology of pathological enhancement purely on the basis of the presence or absence of PVS disease spread; however, this may be used as an extra distinguishing imaging feature (Figs. 2 and 3). Other imaging features including leptomeningeal enhancement, presence of haemorrhagic lesions, spinal cord involvement, specific localisation in CNS or systemic involvement are also helpful for narrowing down the differential diagnosis (Figs. 4, 5, 6 and 7).

**Fig. 8:**
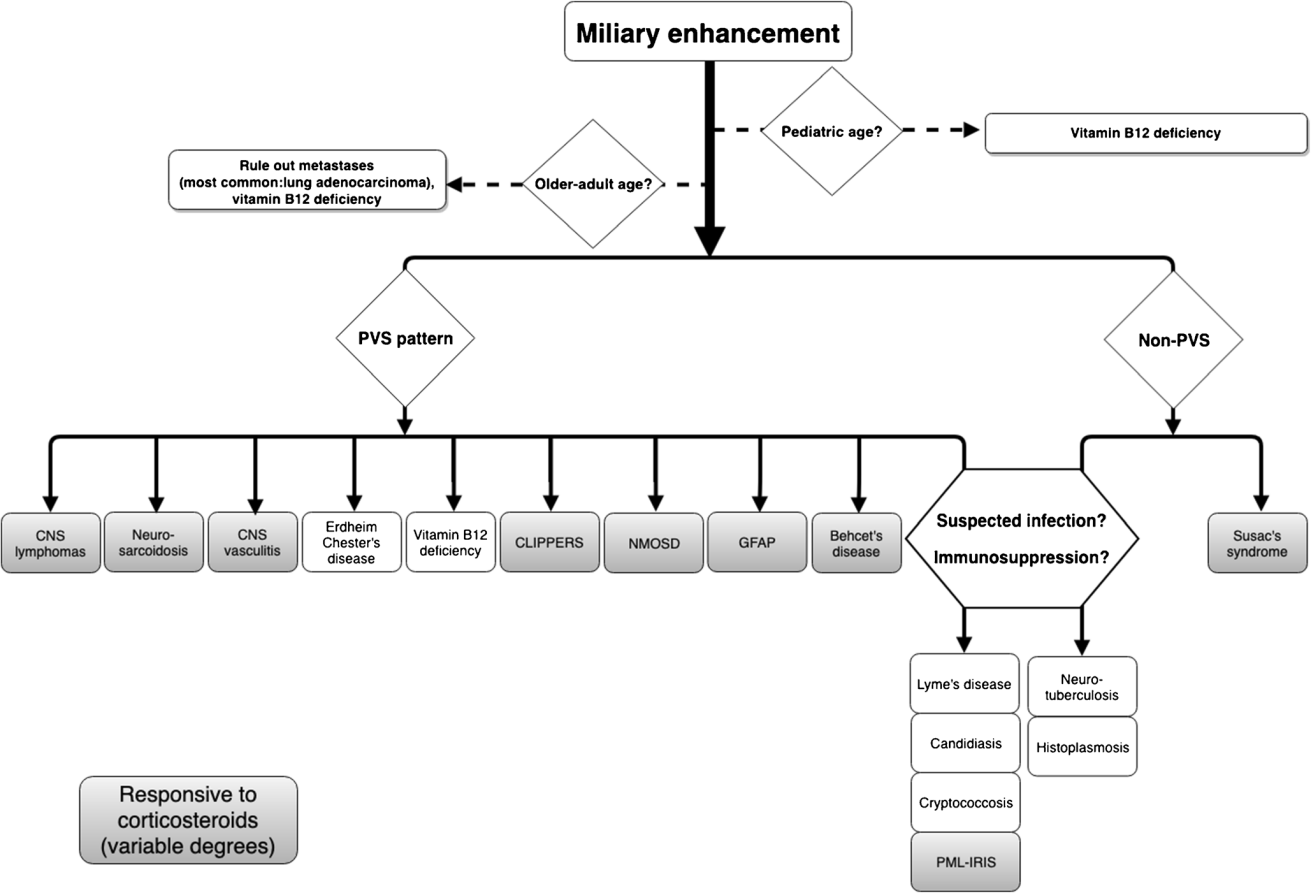
Diagnostic decision tree. Flow chart showing diseases with brain miliary enhancement according to age, PVS distribution patterns and the responsiveness in variable degrees to corticosteroid therapy (PACNS usually needs additional cyclophosphamide therapy)

Finally, in case of follow-up imaging, one could determine the possible response to corticosteroids to further narrow the differential diagnosis (Fig. 8). Brain biopsy should be considered as the last resort in the case of inconclusive clinical and radiological findings.

To demonstrate the role of PVS involvement to narrow down the differential diagnosis, we will discuss the diseases which may show miliary-enhancing brain lesions according to the presence or absence of a PVS distribution pattern of enhancement on post-contrast MRI.

## Miliary-enhancing brain lesions and distribution within PVS

### CNS lymphoma

CNS lymphoma might be either secondary to systemic lymphoma (the most common disease causing a military enhancement pattern) or primary CNS lymphoma (PCNSL), in which the tumour is restricted to the brain, leptomeninges, spinal cord and/or eyes, without involvement of tissues outside the CNS [4].

Aggressive non-Hodgkin lymphoma is the most frequent cause of secondary CNS lymphoma. Approximately two thirds of the patients with cerebral involvement in aggressive non-Hodgkin lymphoma present with leptomeningeal spread, while one third show parenchymal disease [4]. Leptomeningeal spread often affects the cranial nerves, spinal cord and spinal roots, and it may present as cranial or spinal neuropathies [5]. Headache is reported in almost half of the patients with leptomeningeal metastases, and it may be caused by increased intracranial pressure from metastatic obstruction of CSF flow/absorption [4]. The common neuroimaging features of meningeal metastases include arachnoidal, subependymal, dural or cranial nerve enhancement; superficial cerebral lesions; and communicating hydrocephalus [5]. Parenchymal metastases from non-Hodgkin lymphoma often appear as single or multiple enhancing lesions and can be accompanied by leptomeningeal metastases or, sporadically, with a pattern of miliary enhancement characterized by multiple CNS punctate and curvilinear lesions [4, 6, 7]. Cytology of the CSF usually shows malignant cells [8]. CNS lymphoma is very responsive to treatment with corticosteroids, shrinking dramatically within a few days of steroid administration due to their combined cytotoxic (reducing the neoplastic mass) and anti-oedema effects [8].

PCNSL typically affects patients older than 50 years, and male-to-female ratio is approximately 2:1 [2]. CSF examination occasionally shows malignant cells and increased protein concentration [9], and the treatment of choice relies on corticosteroids and chemotherapy [8, 9]. PCNLS can affect both immunocompetent and immunodeficient patients. In immunocompetent patients, PCNSL typically presents as a solitary homogeneously enhancing parenchymal mass, even though multiple lesions are reported in the 20–40% of the cases and ring-like enhancement in 0–13% [4]. Linear enhancement along perivascular spaces is highly suggestive of PCNSL [10]. Most lesions are located in central hemispheric or periventricular cerebral white matter, but superficial location adjacent to the meninges is also common [4]. The brainstem, cerebellum and spinal cord can also be affected [4]. The presence of haemorrhages or calcifications within the tumour is quite a rare finding [11]. On the other hand, immunodeficient patients with PCNSL are often diagnosed with multifocal lesions (30–80% of patients with AIDS-related PCNSL) [4]. Because many lesions exhibit necrotic regions, contrast enhancement is commonly irregular or peripheral, with a ring-like appearance. The basal ganglia and corpus callosum are frequently involved and spontaneous haemorrhage in PCNSL lesions may be more frequent than in immunocompetent patients [4].

Lymphomatoid granulomatosis is a rare angiocentric and angiodestructive lymphoproliferative disease [12]. The granulomatous reaction targets mostly the lungs, but it may also involve the kidneys, skin and the CNS [13]. In the rare case of isolated CNS-lymphomatoid granulomatosis, prognosis is better than systemic lymphomatoid granulomatosis with CNS involvement [12]. On MR imaging, enhancing mass-type lesions may be seen, but multiple miliary and linear enhancements seem to be the more specific findings, as the disease affects preferentially perivascular tissues and the walls of vessels [12]. In addition, leptomeningeal enhancement and dural and cranial nerve enhancement have been reported [12].

### Neurosarcoidosis

Sarcoidosis usually presents as a systemic granulomatous disease with lymphadenopathy, pulmonary infiltration, cardiac and musculoskeletal manifestations and lesions in the skin, liver and eye [14]. Young adults aged 25–45 are most commonly affected, and the incidence depends on age and geography, being higher in non-Caucasian ethnicities and women [14]. About 5% of patients with systemic sarcoidosis may show CNS involvement, whereas isolated neurosarcoidosis, i.e. strictly confined to the CNS, is rare [15]. Diagnostic work-up for systemic sarcoidosis should include serum angiotensin-converting enzyme and human soluble interleukin-2 receptor/CD25, ophthalmological examination, conjunctival biopsy, chest X-ray or CT scan, hand X-ray, bronchial lavage, bronchoscopy and biopsy and lymph node biopsy [14]. Therapy relies on corticosteroids [14].

Central nervous system involvement is very variable, with lesions potentially involving the pachymeninges and leptomeninges (seen in up to 40% of cases), pituitary gland and hypothalamus, cranial nerves and brain parenchyma [15, 16]. Therefore, headache and cranial nerve palsy are common clinical presentations (30–50% of cases), and the facial nerve is very frequently involved, followed by the optic and the vestibulocochlear nerves [14]. Spinal sarcoidosis is relatively common and can manifest with intramedullary, intradural–extramedullary or extradural lesions; cauda equina syndrome; and arachnoiditis [15]. CSF analysis is non-specific, showing increased protein concentration, pleiocytosis and oligoclonal bands. The spectrum of brain MRI abnormalities consists of pachymeningeal involvement, leptomeningeal enhancement (with pituitary, hypothalamic and cranial nerve involvement) and parenchymal lesions (Fig. 4a) [15, 16]. Parenchymal involvement is the most common finding and can manifest in different ways, including extension of leptomeningeal inflammation along PVS, periventricular white matter lesions similar to multiple sclerosis and enhancing masses and nodules [16, 17]. A distribution pattern consistent with PVS involvement has been reported [16, 17], and also in our experience, involvement of the PVS is common (Fig. 2a).

### Erdheim–Chester disease

Erdheim–Chester disease (ECD) is a rare systemic non-Langerhans form of histiocytosis affecting especially the long bones, the lungs and the retroperitoneum [18]. Characteristic X-ray images of long tubular bones show patchy sclerotic lesions with symmetrical involvement of both metaphysis and diaphysis and epiphyseal sparing (Fig. 5e) [18]. Although the age range of patients is wide, ECD typically affects middle-aged or elderly (mean age of 53) males, with a male-to-female ratio of 3:1 [19]. Clinical manifestations are non-specific bone pain and neuroendocrine disturbances, especially diabetes insipidus, which is caused by hypothalamic–sellar masses [19]. Neurological involvement is encountered in less than 50% of cases with the most common localisation including hypothalamic–pituitary axis, brain parenchyma, orbits, meninges and paranasal sinuses [19]. Meningeal involvement occurs in 23% of patients, presenting as single or multiple dural masses that are ‘meningioma-like’, or diffuse pachymeningeal thickening [19]. CSF analysis is non-specific, showing increased protein concentration, low glucose levels and normal cell count. Lumbar puncture is not revealing since ECD histiocytes are rarely present in the CSF and, so far, there are no effective treatment options [20]. Neuroimaging findings include mostly extra-axial, intracranial lesions in the retro-orbital space, pituitary stalk, cerebellopontine angle, choroidal plexus, dura and falx. Spinal cord involvement has also been described [18]. Perivascular distribution has been reported, including extension along the PVS at the level of the basal ganglia, the pons and dentate nuclei, to be possible (Fig. 2b) [2].

### Vitamin B_12_ deficiency

Vitamin B_12_ deficiency can affect both sexes, all economic classes and all ages. High levels of suspicion of inherited vitamin B_12_ metabolism defects should be maintained in children, while elderly individuals might demonstrate the deficiency due to pernicious anaemia or alcohol abuse. In the adults, the condition frequently appears as a result of malabsorption of vitamin B_12_ due to small intestine diseases or therapies which interfere with B_12_ absorption [21, 22]. Also, recreational use of nitrous oxide is spreading rapidly as a cause in young adults [23]. In combination with folic acid, B_12_ is considered crucial in proliferation, maturation and regeneration of neural cells [22]. Serum active B_12_ is the earliest detectable marker that will reveal a deficiency, and methyl malonic acid will increase when vitamin B_12_ storages are depleted [22]. In adulthood, neurological manifestations due to vitamin B_12_ deficiency occur in both sexes across age groups, but the median age range in large series is 70 years to 80 years [22]. The most common clinical finding is symmetric and progressive peripheral neuropathy [21, 22, 24]. CSF analysis could show low levels of transcobalamin II and vitamin B_12_. The administration of vitamin B_12_ supplement may lead to the resolution of the spinal and cranial abnormalities [22]. Cognitive impairment has also been reported as a result of vitamin B_12_ deficiency neurological syndrome, even though replacement therapy is ineffective at this stage [21]. Spinal cord MRI classically shows dorsal column involvement (Fig. 6a, b) and, at a later stage, cord atrophy [24]. Brain involvement might demonstrate extensive areas of a high-intensity signal in the periventricular white matter, and in children with inherited cobalamin-related diseases, brain damage is characterized by white matter loss with delayed myelination [25]. We did not find reports describing miliary PVS enhancement; however, in our experience, this may be seen (Fig. 1).

### Primary and secondary CNS vasculitis

The two CNS vasculitis described with brain miliary enhancement at MRI are primary angiitis of the central nervous system (PACNS) and secondary CNS vasculitis due to chronic graft versus host disease [26, 27].

PACNS, also known as granulomatous angiitis or isolated cerebral vasculitis, leads to a wide spectrum of clinical manifestations including diffuse or focal throbbing headaches, focal or generalized seizures, encephalopathy, brainstem events, stroke-like episodes, aphasia, myelopathy and diffuse neurological dysfunction [26]. Although vessels of any size may be involved, small- and medium-sized ones are most commonly targeted [28]. Males are affected twice as often as females and the onset usually occurs after 40 years of age [26]. Leptomeningeal biopsy can provide a definitive diagnosis, but this procedure is not routinely performed also in light of the fact that a negative biopsy does not rule out the condition [26]. CSF examinations reveal pleiocytosis and increased protein levels [26]. Both clinical and radiological improvements are achieved after high-dose corticosteroids and cyclophosphamide administration [26]. An unremarkable MRI is a very strong argument against the diagnosis of PACNS, and it might serve to exclude it from the differential diagnosis. MRI findings in PACNS include multiple bilateral supratentorial lesions affecting both the grey matter and the white matter, occasionally in combination with diffusion restriction [28]. Leptomeningeal enhancement occurs in 10–15% of the cases [29]. Frequently, segmental vascular lesions may complicate into stenoses, occlusions and infarctions [28], which are often accompanied by white matter hyperintensities [29]. Angiography is considered the gold standard for imaging evaluation, but its sensitivity in detecting PACNS remains limited to the 50–90% range [29]. Angiography may reveal multiple arterial abnormalities such as avascular areas, including uncommon cases of cerebral infarctions along small arterial territories, segmental stenoses, intracerebral aneurysms and narrowing of intracranial vessels eventually alternated by focal dilatations forming a so-called ‘string of beads’ appearance [29, 30]. Aneurysmal rupture leads to either subarachnoid or parenchymal haemorrhage [28]. Salvarani et al. [31] documented spinal cord involvement in about 5% of cases in a study involving 101 patients (Fig. 6d). PVS distribution of miliary-enhancing nodules has been described in PACNS (Fig. 2d) [28].

Secondary vasculitis due to chronic graft versus host disease after haematopoietic stem cell transplantation may also show military enhancement on brain MRI. Terada et al. [27] and Sostak et al. [32] described a brain miliary enhancement along the path of the PVS likely due to the small vessel disruption, either by the direct injury of the endothelial cells or by angiocentric infiltrates composed of various combinations of T lymphocytes, B lymphocytes and histiocytes.

### Chronic lymphocytic inflammation with pontine perivascular enhancement responsive to steroids

Chronic lymphocytic inflammation with pontine perivascular enhancement responsive to steroids (CLIPPPERS) is a pontine-predominant encephalomyelitis, and it was first described in a small group of 8 patients in 2010 as a clinically and radiologically distinct entity [33]. A review by Dudesek et al. [34] described 56 cases worldwide in 2014, with a male predilection and a median age at onset of 52 years (range 16–86 years). Clinical manifestations include ataxia, diplopia, dizziness, nausea, dysgeusia and pseudobulbar affect among others, but there were no reports on systemic symptoms such as weight loss, fever or lymphadenopathy [33, 34]. The pathogenesis remains still unknown, but it could be autoimmune or inflammatory-mediated, and CSF analysis may reveal elevated oligoclonal bands [34].

Recent diagnostic criteria published by Tobin et al. [35] in 2017 include clinical, radiological and pathological features to discriminate CLIPPERS from non-CLIPPERS aetiology, including the presence of (a) homogenous, gadolinium-enhancing nodules without ring enhancement or mass effect predominating in the pons and cerebellum, measuring < 3 mm in diameter and a pattern consistent with PVS involvement (Fig. 2c); (b) marked improvement in abnormal gadolinium enhancement after corticosteroid treatment (Fig. 7); (c) homogenous T2 signal abnormality not significantly exceeding the size of the area of gadolinium enhancement; and (d) spinal cord lesions with similar T2 and gadolinium-enhancing lesions as above [35]. Recently, these diagnostic criteria have been evaluated by Taieb et al. [36] and suggested to consider nodular enhancement as an unusual finding more than a red flag for excluding the diagnosis of CLIPPERS.

In addition to MRI of the brain and the spinal cord, various additional imaging investigations have been performed in CLIPPERS patients and usually revealed no decisive findings. PET-CT of the brain showed no change or minor hypermetabolism of contrast-enhancing areas, considerably less than the findings usually reported in lymphoma [34].

### Neuromyelitis optica spectrum disorders

Neuromyelitis optica spectrum disorders (NMOSD) are inflammatory disorders of the CNS characterized by severe, immune-mediated demyelination and axonal damage predominantly targeting optic nerves and spinal cord, in which the high specificity of aquaporin-4 (AQP4)–immunoglobulin G (IgG) has allowed to recognize other sites of CNS involvement not restricted to the optic nerves and spinal cord as well as diencephalon, brainstem and the brain [37]. Typical MRI findings in NMOSD have been reported as confluent, asymmetric T2 hyperintensities usually distributed in periependymal areas, following the ependymal lining of the lateral, third and fourth ventricles, particularly close to the cerebral aqueduct. The ependymal surfaces of the corpus callosum, diencephalic region and brainstem have also been assumed as typical locations for NMOSD brain lesions [37]. NMOSD scans may occasionally show a ‘cloud-like’ pattern of gadolinium enhancement, with a patchy and inhomogeneous appearance and poorly defined margins; also, periependymal and leptomeningeal enhancements are common [38]. Leptomeningeal enhancement may be thick or linear and is possibly due to AQP4 channel impairment on the pial and subpial surfaces [38]. Pekcevik and Izbudak [39] described a perivascular enhancement pattern during an acute optic neuritis attack in a patient with NMOSD diagnosis, suggesting that PVS enhancement may be seen in NMOSD patients in addition to periependymal and leptomeningeal enhancements. Therapeutic options for acute presentation as well as long-term immunosuppressive treatment include azathioprine, rituximab, immunoglobulins and corticosteroid [37].

### Glial fibrillary acidic protein meningoencephalomyelitis

Glial fibrillary acidic protein (GFAP) meningoencephalomyelitis is a relapsing, autoimmune, corticosteroid-responsive disorder, which sometimes develops as a paraneoplastic autoimmune meningoencephalomyelitis [40]. The detection of GFAP-IgG in serum is a pathognomonic finding [40], while the presence of neuronal, glial or skeletal muscle–specific IgG in serum or CSF aids diagnosis and guides therapy [40, 41]. Fang et al. [41] described a case series of 103 patients with a wide age range (21–73 years, median age of 42 years) and no sex predominance. Clinical manifestations included headache, subacute encephalopathy, optic papillitis, inflammatory myelitis, postural tremor and cerebellar ataxia. Imaging revealed linear perivascular enhancement that resembles the filamentous pial, subventricular and perivascular immunostaining pattern recognisable on murine models [40]. Other enhancement patterns observed less frequently included leptomeningeal and ependymal [40].

The most frequent spinal MRI finding of GFAP autoimmunity is a longitudinally extensive T2-hyperintense lesion, with thin and linear enhancement along the course of the central canal [40].

### Behçet’s disease

Behçet’s disease is a multisystem inflammatory disease of unknown origin, presumably autoimmune in nature and mostly affecting patients from Japan or Mediterranean countries [42]. Onset before the age of 10 and after the age of 50 is rare. The disease shows a male predominance in the Mediterranean region while women are more commonly affected in the Far East [42, 43]. CNS involvement occurs in 10–30% of Behçet’s patients, generally 4–6 years after the disease onset [42, 44]. The clinical presentation of systemic Behçet’s disease shows recurrent oral aphthae (at least 3 times per year in 97% of cases), accompanied by any two of the following: genital ulcers which heal with scarring, erythema nodosum (in 60% of cases), folliculitis, uveitis, skin reactions and hyper-reactivity [42, 45]. Behçet’s disease is responsive to corticosteroid administration [42].

Neuro-Behçet’s disease most commonly presents with bilateral pyramidal signs, hemiparesis and behavioural changes [42]. CSF findings are non-specific, revealing increased protein concentration and pleiocytosis [45]. Typically, the lesions are clustered in the brainstem with a preference for the mesodiencephalic or pontobulbar regions (with typical sparing of the red nuclei), basal ganglia and cerebellar peduncles, and spinal cord involvement is very rare (Fig. 7) [46]. In later stages, brainstem atrophy is a characteristic feature [42]. Furthermore, meningeal involvement, venous thrombosis and delayed intracerebral haemorrhages have been reported [42, 44]. Increased intravascular pressure caused by thrombotic occlusion of the proximal venules and sustained extravasation of erythrocytes via the disrupted walls of small vessels have been proposed as possible causes of the delayed hematoma development typical of Behçet’s disease [44]. This is a different mechanism from arterial haemorrhages, which cause sudden and rapid neurologic deteriorations, or from haemorrhagic transformation of arterial infarctions, also showing an acute onset [44]. Histopathologically, perivascular inflammation has been described in Behçet’s disease [44, 45, 47], but a PVS pattern of enhancement has not yet been reported as an MRI feature. One explanation could be that the absence of endothelial degeneration supports a perivascular inflammatory process as opposed to a vasculitis, and this is not sufficient to cause an evident perivascular enhancement [47]. Given the vascular aetiology of the disease, we decided to consider neuro-Behçet’s disease as part of the group with PVS distribution of enhancement. Moreover, in our case series, we found a CLIPPERS-like infratentorial pattern of miliary enhancement, which can support the thesis of its vascular involvement also in brain imaging.

### Infectious diseases

Different infectious diseases might present with a miliary pattern of cerebral enhancement, and immunocompromised patients are subjects at high risk [2]. The most frequent infectious disorders which present with miliary enhancement are Lyme disease, *Candida albicans*, *Cryptococcus*, progressive multifocal leukoencephalopathy (PML) caused by the opportunistic infection by John Cunningham (JC) virus in immunocompromised patients or patients with MS treated with natalizumab therapy, neurotuberculosis and histoplasmosis, the last two without a PVS pattern of enhancement. CNS infections do not respond to corticosteroid therapy, but they need specific antibiotic or antifungal therapies. As neurotuberculosis and histoplasmosis show an atypical enhancement pattern with respect to all other infectious diseases, they will be described in the following paragraphs.

#### Lyme disease

Systemic borreliosis is considered the most common vector-borne illness caused by spirochetes of the *Borrelia burgdorferi* species [48, 49]. Multiple organ systems may be involved, including the heart, joints, skin, eyes and the CNS, in which case it is referred to as neuro-Lyme disease [48]. Three stages of Lyme disease are identified. First is a prodromal stage with flu-like symptoms and an expanding annular rash, also referred to as erythema migrans (Fig. 5c), 2–30 days after the tick bite. Secondly, both cardiac and neurological symptoms manifest 1–4 months after the bite, followed by arthritis and chronic neurological symptoms which are present after one or more years [49]. The neurological manifestations include peripheral neuropathies, radiculopathies, myelopathies, encephalitis, meningitis, cerebellar signs, cognitive and movement disorders and cranial nerve palsies [49]. Facial nerve palsy is common in neuro-Lyme disease, with bilateral involvement in 50% of cases, and it is considered a rather disease-specific finding (Fig. 4c) [49]. CSF analysis shows reduced glucose levels, increased protein concentration and pleiocytosis, and it is therefore non-specific [48]. Treatment of choice is based on antibiotics [48]. MRI rarely reveals brain lesions, but both meningeal and nerve root involvements are relatively common features [50, 51]. Focal hyperintense T2 multiple sclerosis–like lesions of cerebral white matter have also been reported [50]. Spinal cord involvement by *Borrelia burgdorferi*, on the other hand, is very rare [52]. Cerebral vasculitis in neuro-Lyme disease can affect small-sized as well as medium- and large-sized intracranial arteries, a recent study report on predominant anterior circulation vasculitis in addition to reported PVS involvement [51].

#### *Candida albicans*

*Candida albicans* is a severe fungal infection affecting mostly immunocompromised hosts suffering from HIV and leukaemia, patients with indwelling catheters and premature neonates with significant low birth weight [53]. Reported mortality rates can be as high as 35% of infected cases. *Candida albicans* species demonstrate tropism to a variety of tissues of different organs, including the brain. During the hematologic dissemination phase, known as candidemia, the fungus travels along the bloodstream, with the potential of infecting all the perfused organs [53, 54]. Clinical presentation consists of mucocutaneous candidiasis and systemic candidiasis leading to isolated organ failure or multiorgan failure such as meningitis, endocarditis, peritonitis, osteomyelitis and septic arthritis [54]. Diagnosis can be based on CSF analysis, showing pleiocytosis, decreased glucose and increased protein concentrations and CSF cultures, with positivity estimated around 80% [53]. Management requires antifungal therapy, preferably based on a combination of amphotericin B and flucytosine, and supportive care [54]. Meningitis occurs frequently, and it is classically characterized by headache, neck stiffness and fever. The presence of multiple intracranial (micro) abscesses has also been reported [53]. Mouse studies have revealed adhering and binding of *Candida albicans* to the endothelium of cerebral blood vessels followed by the invasion of the brain parenchyma [55, 56], which might explain intracranial abscesses. Autopsy studies demonstrated that the supratentorial grey–white joint at the level of the basal ganglia is the most affected area in CNS candidiasis [57]. To a lesser extent, *Candida* can cause the development of mycotic aneurysms resulting in intracerebral haemorrhage or subarachnoid haemorrhage, thrombosis in small perforating arterioles resulting in ischaemic stroke, cerebral macro-abscesses and cerebral vasculitis [54].

#### Cryptococcosis

CNS cryptococcosis results from the infection of the CNS by the yeast-like fungus *Cryptococcus neoformans*, and it is the most common fungal infection and the second most common opportunistic infection of the CNS [58]. The strongest risk factor for infection is T cell dysfunction, especially due to HIV infection. The fungal spores in the dehydrated state are small enough to be inhaled and cause an asymptomatic lung infection followed by meningitis [58]. The most common clinical findings in CNS cryptococcal infection are headache, nausea and fever. Less common manifestations are meningism, altered mental state, seizures, visual symptoms and focal neurological deficits [59]. The brain regions most commonly affected are the basal ganglia and meninges. In the basal ganglia, a localized cluster of organisms may develop gelatinous pseudocysts [58]. Cryptococcomas (accumulations of fungi, inflammatory cells and gelatinous mucoid material) appear during the infection and can extend to the parenchyma as focal masses with a tumour-like appearance [59]. Duarte et al. [59] aimed to describe the imaging patterns associated with CSN infection with *Cryptococcus* species in relation to the patient’s immune status (immunocompetent and immunocompromised), describing the dilatation of the perivascular spaces as a common finding in both groups of patients but finding also a perivascular miliary nodule enhancement pattern in one HIV patient with AIDS. Spinal cord infection caused by *Cryptococcus* is rare but might be seen in immunocompromised hosts [60].

#### Progressive multifocal leukoencephalopathy with immune reconstitution inflammatory syndrome (PML-IRIS)

PML is a progressive demyelinating disorder that results from a viral infection of the myelin-producing oligodendrocytes by the JC papovavirus [61]. PML develops from a latent JC virus infection during childhood or early adolescence, which can be reactivated in the setting of immunodeficiency, currently most frequently in multiple sclerosis patients treated with natalizumab [61]. The risk of natalizumab-associated PML increases proportionally to the duration of treatment, with the greatest risk after 2 years of therapy [61]. PML typically results in progressive neurological decline, cognitive impairment, altered mental status and personality changes. Motor and sensory changes also occur, and patients may develop seizures [61]. Spinal cord involvement is extremely rare and only occasionally reported [62].

Current prophylaxis and treatment of PML focus on restoration of immune responses to JC virus infection and interruption of treatment with natalizumab, which might cause an over-reaction of the immune system. The associated immune reconstitution inflammatory syndrome (PML-IRIS) is clinically characterized by new clinical symptoms and/or worsening of existing clinical symptoms mirrored by progressive imaging findings of active inflammation, like contrast enhancement and rapid progression of existing PML lesions showing mass effect with swelling and oedema [63]. PML-IRIS is treated with high-dose glucocorticoids [64]. Since even in early stages PML and PML-IRIS can occur simultaneously, it is crucial to have an early correct imaging-based diagnosis because glucocorticoid therapy for a false-positive PML-IRIS at the stage of productive JC virus infection might lead to PML re-activation and progression [65]. Wattjes et al. [63] described the early PML-IRIS imaging findings in a clinical cohort of 24 subjects with natalizumab-associated PML-IRIS, discovering that, in approximately one third of subjects, one of the earliest signs of PML-IRIS was the presence of small punctuate foci of contrast enhancement with a PVS distribution, peripherally with respect to the main PML lesion. These features were described also by Langer-Gould et al. [66], and this imaging phenomenon is supported by histopathology data demonstrating an inflammatory reaction of B cells as well as CD8^+^ T lymphocytes outside the main PML lesions, particularly in the PVS of patients with PML-IRIS [67].

## Miliary-enhancing brain lesions and distribution outside PVS

### Neurotuberculosis

Tuberculosis can occur at any age, most often in the first three decades of life, with no sex predominance [68]. Frequently reported manifestations of tuberculosis involving the CNS are alterations of consciousness, focal neurological signs, behavioural changes, intracranial hypertension and seizures [68, 69]. CSF examination could show a positive polymerase chain reaction (PCR) and positive culture for TB [68]. Treatment of choice consists of a multidrug approach with isoniazid, rifampicin and pyrazinamide; corticosteroids will not produce an adequate response and are contraindicated [68]. Intracranial tuberculosis can be broadly divided into meningeal and parenchymal patterns of involvement, and any combination of the patterns can occur [70]. Meningeal tuberculosis can be either leptomeningeal or pachymeningeal. Tubercular leptomeningitis is the most common manifestation of CNS tuberculosis, and it is frequently associated with complications like hydrocephalus, ventriculitis and choroid plexitis (Fig. 4b) [70]. Parenchymal tuberculosis, instead, consists of tuberculomas, cerebritis, abscesses, rhombencephalitis and encephalopathy [70]. Parenchymal tuberculomas are the most common form of intracranial parenchymal tuberculosis due to conglomeration of tubercular microgranulomas, which tend to occur at the grey–white matter junction due to arrest of the haematogenous spread caused by a reduction in the calibre of the vessels in that region [70]. Miliary tuberculomas, mostly seen in immunocompromised patients, are usually diffusely scattered in the brain parenchyma, are predominantly located at the grey–white matter junction and occur when the infection reaches the brain through haematogenous spread, usually from a pulmonary focus (Fig. 3a) [69, 70]. No PVS involvement has been reported. Extra-axial spinal tuberculosis could manifest as tuberculous spondylodiscitis (Fig. 5a) [69].

The term ‘miliary tuberculosis’ refers to an uncommon pulmonary manifestation of tuberculosis and represents hematogenous dissemination of uncontrolled tuberculous infection with a poor prognosis (Fig. 5b) [69].

### Histoplasmosis

Histoplasmosis is mainly a pulmonary disease caused by the inhalation of the spores of the fungus *Histoplasma capsulatum* [71]. Patients on immunosuppressive agents, transplant recipients and those affected by either AIDS or haematological malignancies are at risk because of their ineffective immune defences [71]. This fungus is endemic in Northern and Central America, Asia and Africa, which have the highest prevalence, but it also exists in other parts of the world [71]. Usually, this disease is asymptomatic, but older and immunosuppressed patients might present pulmonary findings such as cavitary lesions, granulomatous necrotic mediastinitis and mediastinal fibrosis [72]. In the case of disseminated disease, other organs such as the liver, spleen, bone marrow, lymphoreticular system and, less commonly, the skin are affected [73]. Approximately 5–10% of patients with disseminated histoplasmosis have clinically apparent CNS involvement [71]. Clinical presentation generally reflects the effects of single or multiple space-occupying brain lesions, with headache, focal deficits, altered mental state, stroke-like syndromes and encephalitis [71]. Most lesions clear with antifungal therapy in which posaconazole is most effective [73]. CSF analysis might reveal elevated antibodies against *Histoplasma capsulatum*, and non-specific findings like increased protein concentration, depressed glucose levels and pleiocytosis [71]. Chronic, mainly basilar, meningitis and multiple small ring- or nodular-enhancing lesions throughout the brain and spine are the common findings in CNS involvement (Figs. 3d and 6c) [72]. In the appropriate endemic context, cerebral histoplasmosis should be a diagnostic consideration when imaging studies show multiple brain lesions [73]. There have been no reports of distribution along the PVS.

### Susac’s syndrome

Susac’s syndrome is a rare and presumably autoimmune-mediated disease affecting the small arteries of the brain, inner ear and retina. The clinical triad comprising brain involvement with encephalopathy, hearing loss and branch retinal artery occlusions (Fig. 5d) is considered pathognomonic but is complete only in a minority of patients at disease onset [74]. Other manifestations include headache, confusion, memory loss, dysarthria, behavioural changes, tinnitus and vertigo [75]. Susac’s syndrome mainly affects women aged 20–40 (male-to-female ratio of 1:3) [75]. CSF findings are non-specific, showing pleiocytosis and increased protein concentration [74, 75]. Therapy of choice is corticosteroids; cyclophosphamide can be added as a second-line treatment when corticosteroids fail to produce an adequate response. A review of 27 patients by Susac et al. [76] describes typical involvement of the corpus callosum, the periventricular and subcortical white matter and white matter in the centrum semiovale (100% of cases) (Fig. 3b). Grey matter involvement is less frequent (70% of cases) as well as the presence of enhancing grey and white matter lesions (also 70% of cases). Leptomeningeal enhancement is reported in 33% of cases [76]. The widespread white and grey matter involvement appears not to show a distribution pattern along the PVS. Due to the rarity of Susac’s syndrome, MRI spinal cord involvement has not been well characterized likely due to a lack of routine spinal MRI being performed, but it has been rarely described [77].

## Miliary-enhancing brain lesions and distribution within or outside PVS

### Metastatic disease

Miliary dissemination is a rare manifestation of brain metastases, and it presents as diffuse miliary spread of punctate tumour nodules often with a PVS distribution without invasion of the brain parenchyma, but a non-PVS distribution is also possible (Fig. 3c) [78]. A limited number of autopsy studies confirmed miliary-enhancing lesions in metastatic disease. This rare presentation has been reported mostly in metastatic lung adenocarcinoma [79], but it has been also described in metastatic melanoma [80] and in other types of adenocarcinoma, including breast carcinoma, pancreatic, gastric and salivary gland tumours [78]. Usually, the grey–white matter junction is the preferred site of metastatic carcinomatous cells and metastases may vary in size [79]. Leptomeningeal carcinomatosis resulting from diffuse infiltration in the subarachnoid space also occurs in about 3% of patients with malignancy, usually from the lung, breast or gastric carcinomas [79]. CSF analysis in metastatic disease shows low levels of glucose, increased protein concentration and pleiocytosis and, occasionally, the presence of tumour cells [79]. Treatment of choice should be based on the clinical picture, but it often includes chemotherapy possibly in combination with whole-brain radiotherapy. The administration of corticosteroids might temporarily reduce the perilesional vasogenic oedema and hence relieve symptoms [79].

## Conclusions

The analysis of an MRI scan with brain lesions with a miliary enhancement pattern should always follow a sequential diagnostic algorithm (see Box 1). The review of the medical history together with the identification of disease-specific clinical and lab findings may give important clues to narrow the differential diagnosis (Table 2).

**Table 2:** Clinical information and additional findings

Disease	Sex	Age (years)	Symptoms and signs	Corticosteroid responsiveness	CSF	Additional tests/imaging/information
Neurosarcoidosis	F > M	20–40	Symptoms of increased intracranial pressure due to hydrocephalus and cranial nerve palsy, and systemic symptoms	+	Non-specific	Chest CT, BAL
CNS vasculitis	M > F	Average 50, wide range	Throbbing headache, stroke-like	+	Non-specific	DSA
CNS lymphoma	M > F	Middle age or elderly (> 50)	Headache, symptoms of increased intracranial pressure due to hydrocephalus	+	Malignant cells, increased protein concentration	PET-CT
ECD	M > F	Middle age or elderly (> 50)	Bone pain, neuroendocrine disorders	−	Non-specific	X-rays of long bones
Lyme disease	M = F	Two peaks: 2–15 and 30–55	Erythema migrans, cranial nerve palsy	−	Non-specific	Dermatological exam, serology
Vitamin B_12_ deficiency	M = F	All ages	Anaemia, peripheral neuropathy	−	↓ vitamin B_12_ (uncommon, usually tested in serum)	Spine MRI
CLIPPERS	M = F	Average 50 (wide range)	Gait ataxia, diplopia, dizziness, nausea	+	Rarely oligoclonal bands	Spine MRI, PET-CT
Candidiasis	M = F	Preterm neonates and immunocompromised adults	Meningism, (multi)organ failure	−	CSF culture	Echocardiogram, DSA
Cryptococcosis	M = F	Immunocompromised adults	Meningism, focal neurological deficit	+	CSF culture	Chest CT
PML-IRIS	M = F	Immunocompromised adults	New and/or worsening of existing clinical symptoms of PML (cognitive, motor, sensorial)	+	PCR JC virus	–
GFAP	M = F	Average 40 (wide range)	Meningoencephalomyelitis	+	IgG anti-GFAP	Serum GFAP-IgG
NMOSD	F > M	Average 35 (adolescence − 60)	Unilateral or bilateral optic neuritis, area postrema syndrome, longitudinally extensive myelitis	+	AQP4-IgG (uncommon, usually tested in serum)	Spine MRI
Behçet’s disease	M > F	10–50	Pyramidal signs, behavioural changes	+	Non-specific	DSA
Neurotuberculosis	M = F	Any age, commonly < 30	Focal neurological signs, intracranial hypertension, behavioural changes, seizures	−	PCR, CSF culture	Chest CT
Histoplasmosis	M = F	Immunocompromised adults or at any age in endemic areas	None, cough	−	Antibodies anti-*H. capsulatum* (uncommon, usually tested in serum)	Chest CT
Susac’s syndrome	F > M	20–40	Encephalopathy, hearing loss and visual disturbance	+	Non-specific	Ophthalmological and audiological exam
Metastases	M = F	Elderly	Headache, focal neurological deficit	+/−	Malignant cells	PET-CT

*BAL* bronchoalveolar lavage, *DSA* digital subtraction angiography, *PCR* protein chain reaction, *PML-IRIS* progressive multifocal leukoencephalopathy–immune reconstitution inflammatory syndrome, *ECD* Erdheim–Chester disease, *CLIPPERS* chronic lymphocytic inflammation with pontine perivascular enhancement responsive to steroids, *JC* John Cunningham, *GFAP* glial fibrillary acidic protein, *NMOSD* neuromyelitis optica spectrum disorders

Box 1 Diagnostic checklist

**Table:** 

• **Age** - Childhood: vitamin B_12_ deficiency - Adulthood and geriatric population: CNS lymphoma, metastases and vitamin B_12_ deficiency • **Sociodemographic information** - Behçet’s disease is common in young males from Mediterranean regions, while women are more commonly affected in the Far East - Neurosarcoidosis has a high incidence in non-Caucasian populations - *H. capsulatum* is endemic in Northern and Central America, Asia and Africa • **Medical history and physical examination**: non-neurologic symptoms should raise the suspicion of a systemic disease - Susac’s syndrome (vision and hearing complaints, encephalopathy, headache) - Behçet’s disease (oral and genital ulcerations and uveitis) - ECD (bone pain and neuroendocrine disturbances) - Sarcoidosis (pulmonary, mediastinal, cardiac, musculoskeletal manifestations) • **Immunocompromised patient or clinical signs suggestive for infection**: consider CSF - PML-IRIS is highly suspected in PML subjects after discontinuation of natalizumab • **Imaging findings** - *Localisation*: CLIPPERS and neuro-Behçet’s disease usually present with an infratentorial localisation - *Enhancement with or without PVS pattern* (see Table 1) - *Haemorrhagic lesions*: typical imaging feature of CNS vasculitis, *Candida albicans* infection and Behçet’s disease - *Meningeal enhancement*: common in CNS lymphoma, adenocarcinoma metastases, neurosarcoidosis, NMOSD and infectious diseases (neurotuberculosis, Lyme disease, histoplasmosis, *Candida* infection) - *Spinal cord involvement*: frequent imaging feature in neurosarcoidosis, vitamin B_12_ deficiency, CLIPPERS and GFAP meningoencephalomyelitis. Neurotuberculosis and *Candida* infection may present with osseous vertebral involvement • **Positive response to corticosteroids** - CLIPPERS - CNS lymphoma - CNS vasculitis - NMOSD - GFAP meningoencephalomyelitis - Behçet’s disease - Neurosarcoidosis - PML-IRIS - Susac’s syndrome • **Biopsy of non-brain tissue** (e.g. lymph nodes)	

An accurate medical history, including socio-demographic findings and physical examination, is the first step to identify the diseases with a multiorgan involvement, such as Susac’s syndrome, Behçet’s disease, ECD, sarcoidosis and CSN lymphoma. The latter is probably the most common cause of cerebral miliary enhancement in the Western world. The classic triad of Behçet’s disease consists of oral and genital ulcerations and ocular manifestations, and it is more common in the Middle East and East Asian countries [42]. Susac’s syndrome is clinically characterized by two out of the three features of its clinical triad: acute or subacute encephalopathy, bilateral sensorineural hearing loss and branch retinal arterial occlusions [76]. ECD affects multiple organs like long bones, the lungs, the retroperitoneum and the neuroendocrine system [18]. The lungs and the skin are primarily involved in sarcoidosis [14] in combination with pulmonary, mediastinal, cardiac, musculoskeletal and eye involvement [14].

\In the paediatric population, work-up for inherited vitamin B_12_ metabolism defects should be performed. Vitamin B_12_ deficiency should also be sought in elderly people with an imbalanced diet, pernicious anaemia and alcohol abuse. In the adult and elderly populations, metastases should always be ruled out from the differential diagnosis before considering more rare diseases. Lung adenocarcinoma is the most frequent tumour histotype presenting with cerebral metastases with a miliary enhancement pattern [79], followed by breast carcinoma, pancreatic tumour and salivary gland tumour [78].

The presence of an underlying infectious disease should be considered in immunocompromised patients or in the case of suggestive clinical signs and symptoms such as fever and cutaneous rash. The identification of disease-specific clinical and lab findings will help to narrow down the possible differential diagnoses (Table 2). Evidence of CSF positivity to specific antibodies, the detection of pathogens through CSF culture or PCR would point towards a diagnosis of CNS infection, although a high level of suspicion should be maintained in the case of Lyme disease as the CSF findings may be non-specific [48].

After ruling out age-specific differential diagnoses, imaging features like PVS enhancement pattern, preferential localisation (infratentorial and supratentorial), haemorrhagic lesions, leptomeningeal enhancement and spinal cord involvement are helpful in the diagnostic assessment (Table 3).

**Table 3:** Radiological findings

Disease	PVS pattern	Localisation	Leptomeningeal enhancement	Haemorrhages	Spinal cord involvement
Neurosarcoidosis	+	Supratentorial and infratentorial	+	Rare	+
CNS vasculitis	+	Supratentorial and infratentorial	+	+	Rare
CNS lymphoma	+	Supratentorial and infratentorial	+	Rare in immunocompetent	+
ECD	+	Supratentorial and infratentorial	+	−	Rare
Lyme disease	+	Supratentorial and infratentorial	+	−	+
Vitamin B_12_ deficiency	+	Supratentorial and infratentorial	−	−	+
CLIPPERS	+	Infratentorial	−	−	+
Candidiasis	+	Supratentorial and infratentorial	+	+	− (vertebral bodies)
Cryptococcosis	+	Mostly supratentorial	+	−	Rare (immunocompromised)
PML-IRIS	+	Supratentorial and infratentorial	−	−	−
GFAP	+	Supratentorial and infratentorial	+	−	+
NMOSD	+	Supratentorial and infratentorial	+	−	+
Behçet’s disease	+	Infratentorial	+	+	−
Neurotuberculosis	−	Supratentorial and infratentorial	+	−	− (vertebral bodies)
Histoplasmosis	−	Supratentorial and infratentorial	+	−	+
Susac’s syndrome	−	Mostly supratentorial (100% corpus callosum)	+	−	Rare
Metastases	+/−	Supratentorial and infratentorial	+	+/−	+

*PML-IRIS* progressive multifocal leukoencephalopathy–immune reconstitution inflammatory syndrome, ECD Erdheim–Chester disease, *CLIPPERS* chronic lymphocytic inflammation with pontine perivascular enhancement responsive to steroids, *GFAP* glial fibrillary acidic protein, *NMOSD* neuromyelitis optica spectrum disorders

CLIPPERS and Behçet’s disease commonly display an infratentorial distribution, while Behçet’s lesions typically involve the mesodiencephalic or pontobulbar regions, basal ganglia, internal capsule and peduncles with sparing of the red nucleus [46], CLIPPERS lesions involve the pons and extend to the spinal cord, towards the basal ganglia and cerebellum [33]. Moreover, symmetrical T2 hyperintensities in the dentate nuclei and peridentate regions should raise the suspicion of ECD.

The distribution pattern of the enhancement should be investigated to establish whether it is consistent with PVS enhancement or not. The presence of PVS enhancement pattern has a highly positive predictive value, but if not detected, the whole differential diagnoses should be considered. In this setting, Behçet’s disease deserves a specific mention, because the PVS enhancement pattern in MRI case series of Behçet’s disease has never been reported, even though analyses of pathological specimens have proven involvement of vessels’ walls, making it theoretically possible to mimic PVS pattern in brain imaging.

If haemorrhagic lesions are present, PACNS, *Candida albicans* and Behçet’s disease should be considered primarily, as they cause cerebral vasculitis which may result in intracerebral or subarachnoid haemorrhage [28, 44, 54].

Leptomeningeal enhancement is frequent in—but not limited to—CNS lymphoma, adenocarcinoma metastases, neurosarcoidosis, NMOSD and infectious diseases (neurotuberculosis, Lyme disease, histoplasmosis, *Candida albicans*) that present with meningitis [49, 53, 70, 71]. Leptomeningeal involvement is not reported for vitamin B_12_ deficiency and PML-IRIS, but if this imaging feature is present, Susac’s syndrome, Behçet’s disease, PACNS, ECD and GFAP meningoencephalomyelitis should also be maintained in the differential diagnosis [4, 15, 40, 76].

Spinal cord involvement is frequent in NMOSD as well as in neurosarcoidosis, vitamin B_12_ deficiency, CLIPPERS, histoplasmosis and GFAP meningoencephalomyelitis [15, 24, 34, 40]. Myelitis is not a conclusive finding since it has also been reported for the other pathologies described in this review, even though in the latter cases, spinal cord involvement would be rare and not typical. Neurotuberculosis and *Candida albicans* may be present with osseous vertebral involvement, but spinal cord is usually spared [69, 81].

Limitations with regard to the detection of these small miliary-enhancing lesions mainly lie within slice thickness and contrast dose used. Applying double- or triple-dose gadolinium and 1-mm-thin slice post-contrast 3D T1-GRE or 3D FLAIR sequences may increase the detection rate of the small enhancing nodules or leptomeningeal enhancement [82].

Applying diffusion-weighted imaging (DWI) may be useful to differentiate between lesions showing diffusion restriction compared to T2 shine through, for example CNS lymphoma or small ischaemic lesions in vasculitis compared to Susac’s syndrome.

Unfortunately, for lesions smaller than 3 mm, both single- and multivoxel ^1^H-magnetic resonance spectroscopies are not feasible. Other advanced MR techniques such as perfusion studies with arterial spin labelling and diffusion tensor imaging are often inconclusive unless a bigger and measurable lesion is present.

In the case of ongoing uncertainty regarding the definitive diagnosis, response to empirical immunosuppressive medication with corticosteroids could further drastically narrow down the differential diagnosis (refer to the flow chart, Fig. 8). With systemic diseases remaining within the differential diagnosis, biopsy of non-brain tissues, such as lymph nodes, should therefore always be considered before considering to proceed to brain biopsy.
